# Bovid microRNAs involved in the process of spermatogonia differentiation into spermatocytes

**DOI:** 10.7150/ijbs.38232

**Published:** 2020-01-01

**Authors:** Chuanfei Xu, Mujahid ali Shah, TserangDonko Mipam, Shixin Wu, Chuanping Yi, Hui Luo, Meng Yuan, Zhixin Chai, Wangsheng Zhao, Xin Cai

**Affiliations:** 1 Key Laboratory of Qinghai-Tibetan Plateau Animal Genetic Resource Reservation and Utilization, Sichuan Province and Ministry of Education, Southwest Minzu University, Chengdu 610041, Sichuan, China.; 2 School of Life Science and Engineering, Southwest University of Science and Technology, Mianyang 621010, Sichuan, China.

**Keywords:** cattleyak, male infertility, microRNA profiling, spermatogenesis, STA-PUT

## Abstract

The male infertility of cattleyak resulted from spermatogenic arrest has greatly restricted the effective utilization of the heterosis from crossbreeding of cattle and yak. Based on our previous studies, the significant divergences of the transcriptomic and proteomic sequencing between yak and cattleyak prompt us to investigate the critical roles of microRNAs in post-transcriptional regulation of gene expression during spermatogenesis. TUNEL-POD analysis presented sharply decreased spermatogenic cell types and the increased apoptotic spermatogonia in cattleyak. The STA-PUT velocity sedimentation was employed to obtain spermatogonia and spermatocytes from cattle, yak and cattleyak and these spermatogenic cells were verified by the morphological and phenotypic identification. MicroRNA microarray showed that 27 differentially expressed miRNAs were simultaneously identified both in cattleyak vs cattle and in cattleyak vs yak comparisons. Further analysis revealed that the down-regulation of bta-let-7 families, bta-miR-125 and bta-miR-23a might impair the RA-induced differentiation of spermatogonia. Target gene analysis for differentially expressed miRNAs revealed that miRNAs targeted major players involved in vesicle-mediated transport, regulation of protein kinase activity and Pathways in cancer. In addition, spermatogonia transfection analysis revealed that the down-regulation of bta-miR-449a in the cattleyak might block the transition of male germ cells from the mitotic cycle to the meiotic program. The present study provided valuable information for future elucidating the regulatory roles of miRNAs involved in spermatogenic arrest of cattleyak.

## Introduction

Cattleyak (CY), the hybrids between cattle (♂, CL) and yak (♀, YK), exhibit much higher performance than YK in such economic traits as meat and milk production. However, the infertility of F1 CY males resulted from spermatogenic arrest has greatly restricted the effective utilization of heterosis from the hybrids and cultivation of the new breeds inherited the excellent adaptive and productive traits from cattle and yak. Therefore, an understanding of the mechanism of male infertility in CY is a critical step for breeding and improvement of YK. Spermatogenesis is a complex and highly organized process of continuous germ cell differentiation which contains three principal phases: mitotic proliferation of spermatogonia, meiosis of spermatocytes and spermiogenesis of haploid spermatids. In non-primates, spermatogenesis is based on the renewal and differentiation of Spermatogonial Stem Cells (SSCs), followed by the proliferation of sequencial types A_pr_, A_al_, A_1-4_, intermediate, and type B spermatogonia which gives rise to spermatocytes 1. The isolation and identification of male germ cells establish the foundation for molecular mechanism research involved in these three main phases of spermatogenesis. To date, varied protocols have been applied to the isolation of mixture cell types by positive or negative select according to the physical or characteristics such as cell surface markers, adhesivity, motility and buoyant density 2. Magnetic-activated cell sorting (MACS) provided an efficient and fast approach for the isolation of CD9^+^ SSCs from the goat testis compared to differential plating and Discontinuous Percoll Centrifugation (DPC) 3. GPR125^+^ spermatogonia was also successfully isolated from adult human by MACS 4. Fluorescent-activated cell sorter (FACS) separation was the most suitable method for the isolation of spermatids form obstructive azoospermic men testis compared with velocity sedimentation under unit gravity (VSUG) combined with DPC 5. Additionally, the STA-PUT velocity sedimentation allowed for the separation of spermatogonia, pachytene spermatocytes and round spermatids in mice 6,7 and human 8. Recently, we have also conducted the analogous research to obtain spermatogonia and spermatocytes from CL, YK and CY by STA-PUT velocity sedimentation with over 85% purity 9.

Over the past decades, many studies have been done to investigate the mechanisms of spermatogenic arrest of CY based on morphological anatomy, histological observation, cytogenetic analysis and gene expressions involved in a specific developmental stage in spermatogenesis 10-12. We have analyzed the transcriptomic and proteomic differentiation between YK and CY to screen differentially expressed (DE) genes and proteins involved in spermatogenic arrest of CY 13-15. Although much work has been implemented to investigate the mechanisms of CY infertility, little is yet known about post-transcriptional regulations controlling mitotic proliferation of spermatogonia and meiosis of spermatocytes. Recently, a number of researches have reported that microRNAs (miRNAs), a class of endogenous non-coding small RNA molecules, regulate gene expression at a post-transcriptional level by binding to the 3' untranslated regions (3'UTR) of target mRNAs for degradation or inhibiting translation 16-18 and play different roles in such biological processes as cell proliferation 19, differentiation 20 and apoptosis 21. The down-regulation of miR-135a was revealed to activate *FoxO1* gene involved in cellular proliferation, which resulted in the decreased number of SSCs and failure of spermatogonial stem cell maintenance in cryptorchid testes 22. MiR-469 repressed the expression of *TP2* and *Prm2* at the translation level with minor effect on mRNA degradation, which was essential in pachytene spermatocytes and round spermatids for their timely translation to attain mature sperm at later times of spermiogenesis 23. In addition, over-expression of miR-34c promoted meiosis by suppressing the expression of *Nanos2* and leading the up-regulation of genes associated with meiosis including *Nanos3*,* Scp3* and *Stra8* in mouse spermatogenesis 24. All of these studies demonstrated that miRNAs play a critical role in regulating gene expression involved in spermatogenesis. Nevertheless, miRNA profiles and their regulation roles in spermatogenic cells of CL, YK and CY remain to be defined.

Here, we obtained spermatogenic cells containing spermatogonia and spermatocytes from CL, YK and CY by using STA-PUT velocity sedimentation. Then, we identified the DE miRNAs and their target genes in spermatogenic cells. The further characterization of these miRNAs was conducive to reveal their regulatory roles involved in spermatogenic arrest of CY and the miRNAs together with their target genes might serve as effective molecular markers in resolving the problems of spermatogenic arrest of CY in the future.

## Material and methods

### Ethics statement

Sample collection was carried out under the license in accordance with the Guideline for Care and Use of Laboratory Animals of China and all animal procedures were approved by the institution Review Board of Southwest Minzu University and Southwest University of Science and Technology.

### Cell apoptosis analysis of testicular structure for YK and CY

Testes of healthy Simmental bull, Maiwa yak and cattleyak (n=3) aged 24 months were collected from Qingbaijiang slaughter houses in Chengdu, Sichuan province. Testis of each animal obtained was placed in an insulated container at 4 ℃ and then transported to laboratory for subsequent experiment after 1h.

TUNEL-POD was employed to examine the cell apoptosis of testicular samples from YK and CY. The apoptotic DNA fragmentation was detected based on TdT-catalyzed incorporation of fluorescein-labeled nucleotides to free 3'-OH ends of DNA in a template-independent manner by using a TUNEL-POD assay kit according to the manufacturer's instructions (Experiment Centre of Biomedicine, ChengDu, China). In the present study, three different segments of each testis of YK and CY were collected for slices by fine-scale dissection after epididymis, fat, connective tissues and fascia were removed. Micrographs of *In situ* DNA fragmentation in each resulting segment were taken by a microscope equipped with a digital photo-camera. The TUNEL-positive cells in seminiferous tubules were considered as cells with dark brown nuclei. Student's T-test was conducted for the significant analysis of cell apoptosis between the testis of YK and CY.

### Isolation and identification of spermatogenic cells in CL, YK and CY

The protocol for the isolation of spermatogenic cells from bovine testicular tissue (12 g for each time) was performed by employing STA-PUT apparatus as described previously 9. The pooled cell fractions were added 600 µL TRIzol reagent (Invitrogen, CA, USA) according to the manufacturer's instructions for next RNA extraction.

Total RNA was enriched from the freshly isolated spermatogenic cells containing spermatogonia and spermatocytes from CL, YK and CY using the TRIzol reagent (Invitrogen, Carsbad, CA, USA) according to the manufacturer's instruction. RT-PCR was performed for the chosen marker genes including *CD9, UCHL1, RET, Tesmin, SYCP1* and *SYCP3* as described previously 9. The primer sequences of these genes were listed in Supplementary Table S1. *GAPDH* was used as internal reference.

### MiRNA microarrays

The integrity of total RNA extracted from spermatogonia and spermatocytes of CL (n=3), YK (n=3) and CY (n=3) was evaluated by Agilent Bioanalyzer 2100 (Agilent Technologies, Santa Clara, CA, US) and up to the requirement of Affymetrix miRNA 4.0. The RNA labeled with FlashTag Biotin HSR was stained after hybridization and the slides were immediately scanned using GeneChip® Scanner 7G (Affymetrix, Santa Clara, CA, US). Command Console Software 3.2 (Affymetrix, Santa Clara, CA, US) was used to analyze array images to get raw data and then Expression Console (Affymetrix, Santa Clara, CA, US) offered RMA+DABG (Robust Multi-array Average plus Detection Above the Background) normalization. To define the differential expression profiles within the different variants, a one-way Anova was performed in the SAS software. The significant DE miRNAs were selected according to |log_2_ (Fold change)| ≥ 1 and P-value < 0.05.

### Prediction and pathway analysis of target genes for DE miRNAs

Target prediction was performed using MiRNA Targets Prediction (v2.0 beta) in term of the following criteria: targets located in the 3'-UTR region, seed length of at least 7 base pairs and P < 0.05. Gene Ontology (GO) and Kyoto Encyclopedia of Genes and Genomes (KEGG) enrichment were applied to classify the target genes of DE miRNAs based on their biological functions. These two methods firstly calculated the target gene numbers for each term or pathway after comparing to a genome background and then the hypergeometric test was used to filter the significantly enriched terms or pathways 25.

### Cell transfection

Spermaogonia of YK were transfected with bta-miR-34c mimics and bta-miR-34c inhibitors in a 48-well plate which is purchased from Genepharma Co. (Shanghai, China), and scrambled oligonucleotides (NC) as control. Bta-miR-34c mimics/inhibitors were diluted to 10 ng in 63 µL Opti-MEM (Invitrogen) reduced serum medium, then the mixture of 63 µL Opti-MEM and 1 µL Lipofectamine® 3000 was added directly into the diluted bta-miR-34c mimics/inhibitors. After incubated for 5 min at room temperature, 120 µL transfection medium was mixed with the isolated spematogonia and incubated at 37 °C, 5% CO_2_. The transfection medium was replaced by fresh growth medium after incubation for 6 h, and the spermatogonia were collected after 48 h and lysed with 600 µL TRIzol reagent (Invitrogen, CA, USA) according to the manufacturer's instructions.

### qPCR validation for the expression of miRNA and their target genes

Stem-loop reverse transcription and quantitative real-time PCR (qPCR) with SYBR Green were performed to validate the expression of miRNAs and their target genes as described previously 25. The primer sequences of miRNAs and genes used for qPCR were listed in Supplementary Table S1. *U6* and *β-actin* were used as internal reference for miRNAs and genes, respectively.

## Results

### Cell apoptosis presented in YK and CY testis

**Figure 1:**
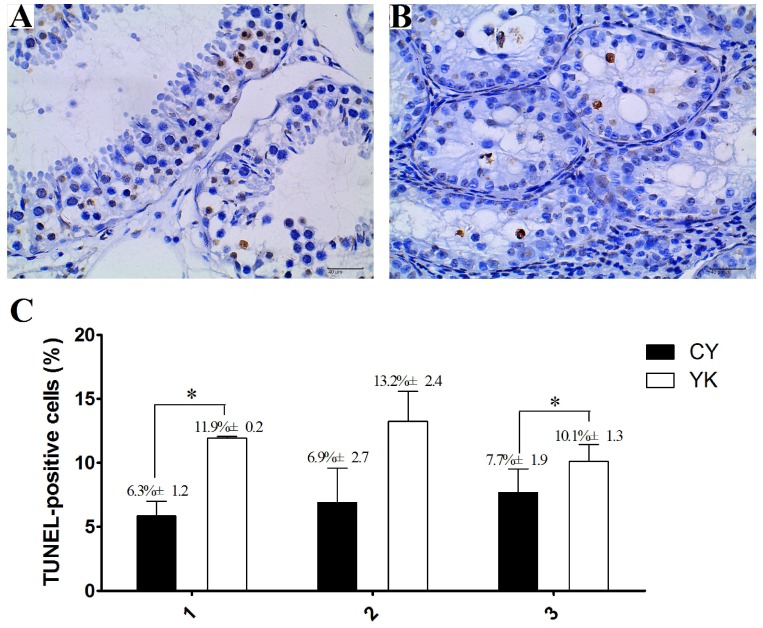
** Cell apoptosis analysis of YK (A, C) and CY testis (B, C).** A dark brown nucleus represented the TUNEL-positive cells in seminiferous tubules and three repeats for the analysis of cell apoptosis were conducted as X axis represented. Quantitative data are means±SD for three repeats of each group. *P < 0.05 versus corresponding control (T-test). Bars = 40 µm.

The obvious decrease of cellularity was observed in seminiferous tubules of CY compared with YK. As shown in Fig. 1A, abundant spermatogenic cells at stage-specific development were present in YK testis including round and lengthened spermatids, while the main type of germ cells was spermatogonia in CY and very fewer spermatocytes and no round or lengthened spermatids were observed (Fig. 1B). The mean number of apoptotic cells in YK was significantly higher than those from CY (Fig. 1C). The most germ cells undergoing normal apoptosis were meiotic primary spermotocytes in YK, while almost all the apoptotic germ cells were spermatogonia in CY and the apoptosis was probably aggravated in the spermatocytes of CY. Therefore, TUEL-POD analysis presented sharply decreased spermatogenic cell types and the increased apoptotic spermatogonia in CY.

### Isolation and identification of the spermatogonia and spermatocytes from CL, YK and CY

**Figure 2:**
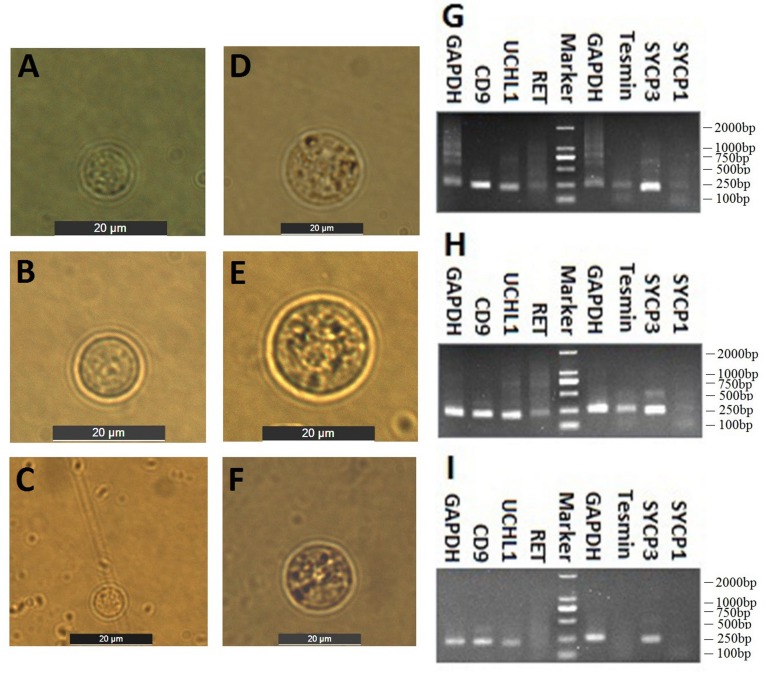
** Isolation and identification of spermatogonia and spermatocytes.** Inverted microscope showed the spermatogonia **(A-C)** and spermatocyte **(D-F)** from CL, YK and CY. Scale bars = 20 µm. RT-PCR revealed RT-PCR analysis showed that *CD9* and* UCHL1* were expressed in the isolated spermatogonia, while* Tesmin* and *SYCP3* were expressed in the isolated spermatocytes from CL, YK and CY **(G-I)**.

Spermatogenic cells containing spermtogonia and spermatocytes were isolated from CL, YK and CY by STA-PUT apparatus via the velocity sedimentation. The isolated cell types were identified according to the morphological and phenotypic characteristics. The diameter of spermatogonia collected from CL and YK were around 10 µm and 15 µm, respectively, which were larger than those from CY (around 8.5 µm) (Fig. 2A-C). Meanwhile, spermatocytes isolated from CL and YK were around 20 µm in diameter, which were also larger than those from CY (around 18 µm) (Fig. 2D-F). To further verify the phenotypic features of the isolated cells, expression of various marker genes (*CD9, UCHL1* and* RET* for spermatogonia; *Tesmin, SYCP1* and *SYCP3* for spermatocytes) were detected. RT-PCR analysis showed that *CD9* and* UCHL1* were expressed in the isolated spermatogonia, while* Tesmin* and *SYCP3* were expressed in the isolated spermatocytes (Fig. 2G-I). Since STA-PUT isolation primarily eliminated somatic cells and round spermatids, an over 90% purity of germ cells comprising spermatogonia and primary spermatocytes were obtained from CL, YK and CY for further miRNA microarrays, in which 34.7%, 33.3% and 26.3% of spermatocytes accounted for the samples from CL, YK and CY, respectively (Supplementary Fig. S1).

### DE miRNAs in the spermatogonia and spermatocytes of CL, YK and CY

**Figure 3:**
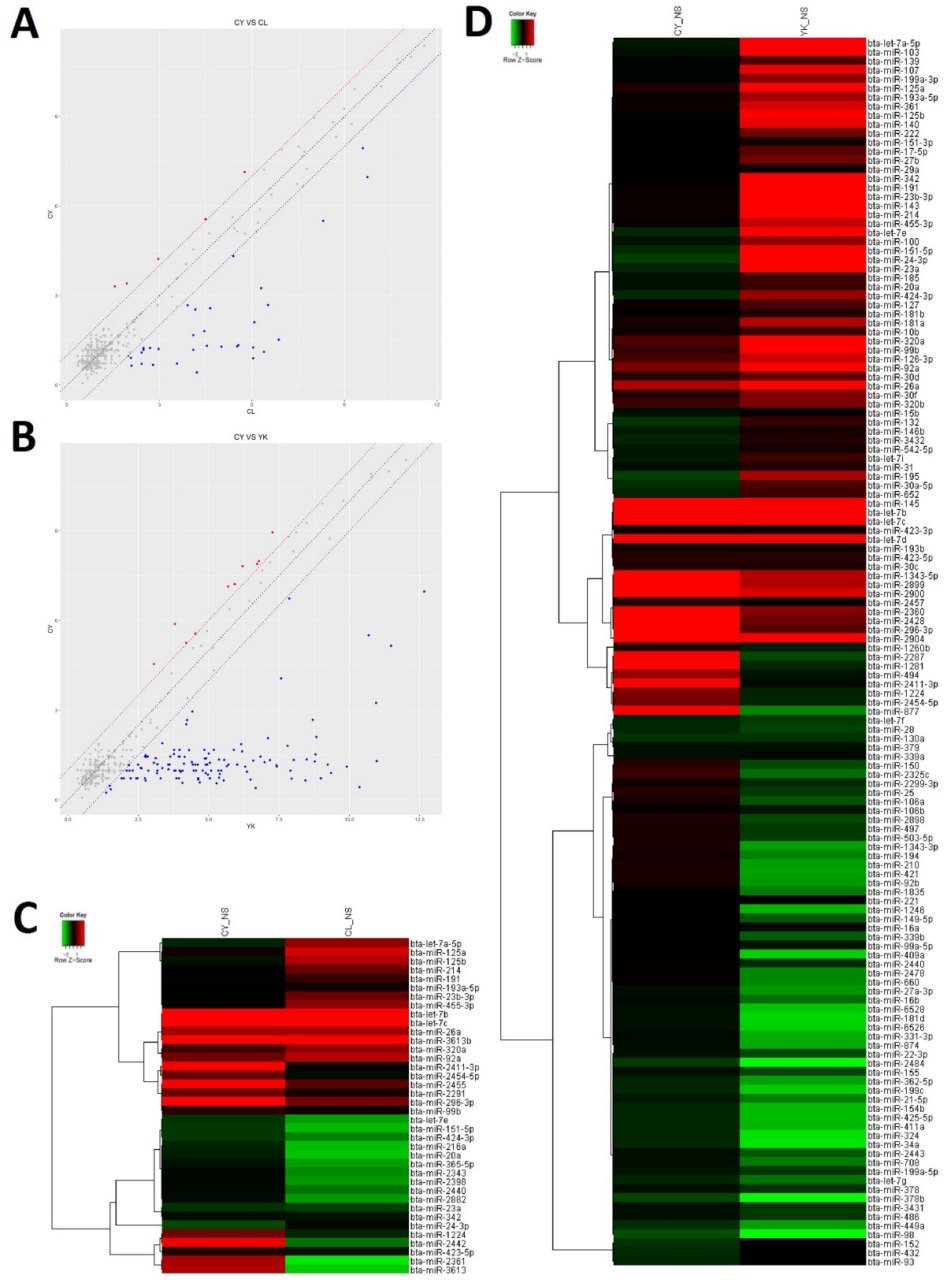
** The differential expressed miRNAs in CY compared with CL and YK.** Scatter plot revealed the overall distribution of DE miRNAs in CY vs CL **(A)** and CY vs YK **(B)**, in which red and blue points represented up-regulated and down-regulated miRNAs, respectively, with fold change ≥ 2. Heat-map was constructed for DE miRNAs in CY vs CL **(C)** and CY vs YK **(D)**.

In order to gain insight to the possible roles of miRNAs involved in the spermatogenic arrest of cattleyak during the differentiation of spermatogonia into primary spermatocyte, miRNA microarrays were performed to investigate the DE miRNA profiles of the spermatogonia and spermatocytes isolated from CL, YK and CY. There were totally 147 DE miRNAs (≥2-fold changes) identified from the spermatogonia and spermatocytes of CY in comparison with those from YK and CL (Supplementary Fig. S2). Among these 147 DE miRNAs, 38 were obtained between CY and CL (Supplementary Table S2), 136 were obtained between CY and YK (Supplementary Table S3) and 27 miRNAs were found to be commonly differentially expressed in pairwise comparison between CY and CL or YK (Table 1), suggesting that these miRNAs may be likely to play important roles during the differentiation of spermatogonia into primary spermatocyte in cattleyak. The ArrayExpress number for public access of DE miRNAs in CY compared with CL or YK was E-MTAB-6964 (http://www.ebi.ac.uk/arrayexpress). For the 38 spermatogenic DE miRNAs between CY and CL, 33 miRNA were identified to be significantly down-regulated and 5 were up-regulated in CY (Fig. 3A, C). Among the 136 spermatogenic DE miRNAs between CY and YK, 126 miRNAs were found to be significantly down-regulated and 10 were up-regulated in CY (Fig. 3B, D). Furthermore, among the common 27 DE miRNAs between CY and CL or YK, 25 were revealed to be commonly down-regulated and only 2 were commonly up-regulated in CY.

### Prediction, GO and KEGG enrichments of target genes for DE miRNAs

To identify the potential roles of DE miRNA in the spermotogonia and spermatocytes from CL, YK and CY, target gene prediction was performed using MiRNA Targets Prediction (v2.0 beta). Target genes for the 38 DE miRNAs between CY and CL were shown in Supplementary Table S4, and for the 136 DE miRNAs between CY and YK were shown in Supplementary Table S5. The results showed that all the miRNAs had multiple target genes possessing a wide range of diverse function and a specific gene could been targeted by multiple miRNAs.

**Figure 4:**
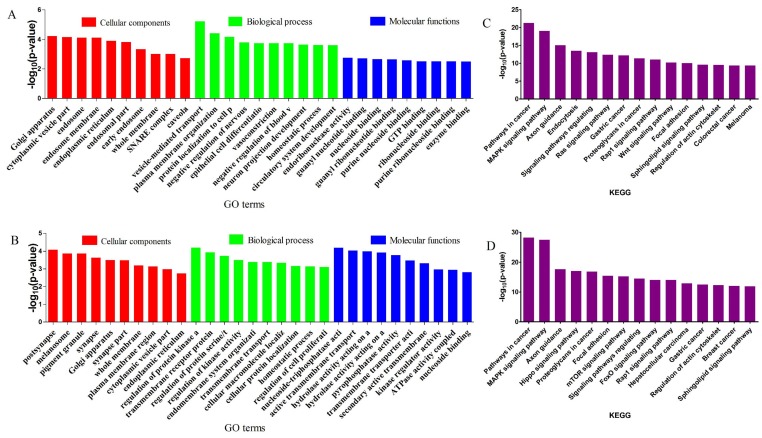
** GO and KEGG enrichment of target genes for DE miRNAs.** The top 5 items of GO enrichment of target genes for DE miRNAs in CY vs CL **(A)** and CY vs YK **(B)** were based on biological process, molecular function and cellular component, respectively. The top 10 pathways of KEGG enrichment of target genes for DE miRNAs in CY vs CL **(C)** and CY vs YK **(D)** were ranked according to decreased -log10 of p values listed on the y-axis.

**Figure 5:**
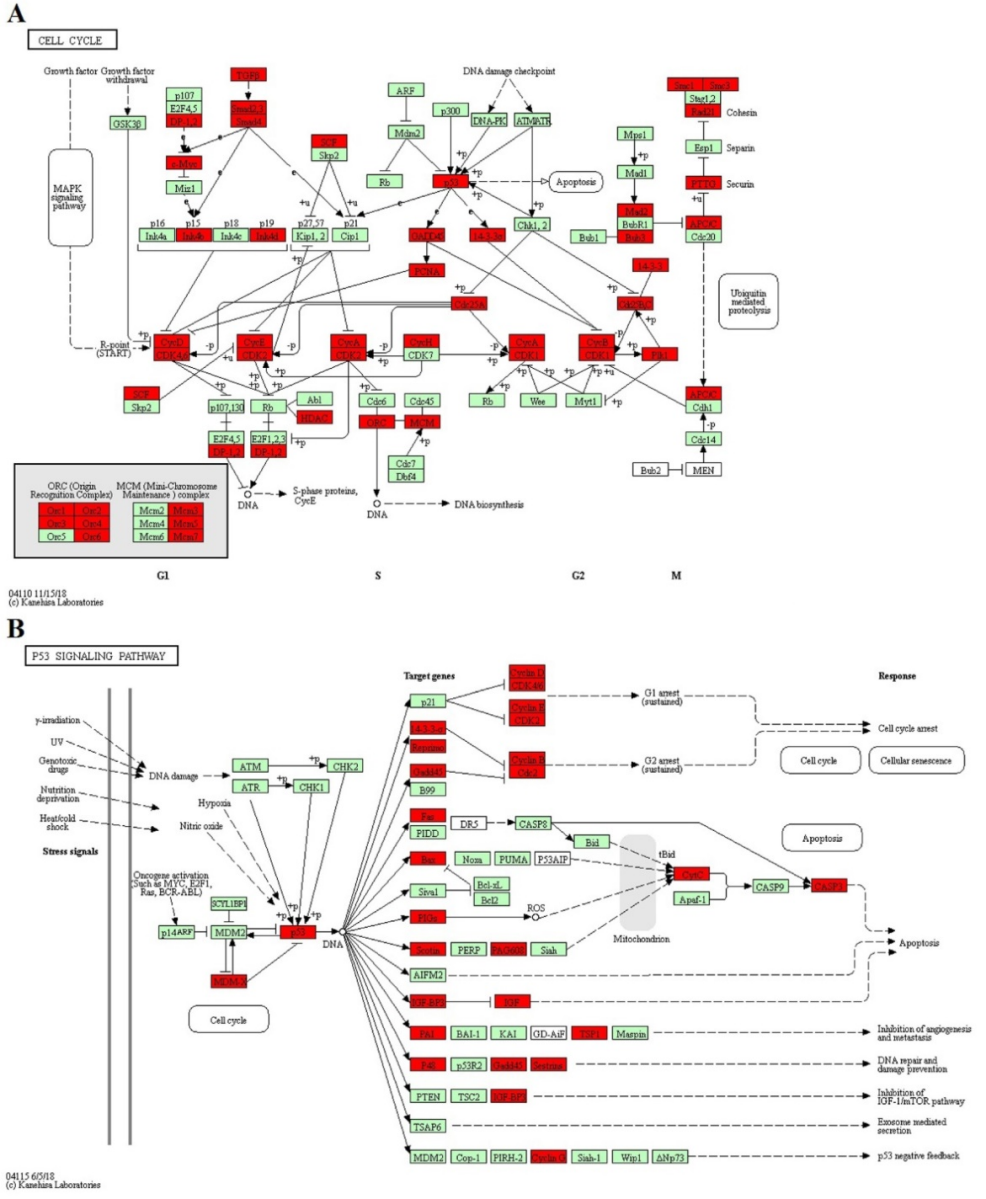
** The KEGG pathway: CELL CYCLE (A) and P53 SIGNALING PATHWAY (B).** Red represented the target genes of DE miRNA and Green represented the specific genes of* bos taurus.*

GO enrichment and KEGG pathway analysis were applied to classify the target genes for DE miRNAs based on their biological functions (Fig. 4). As a result, a total of 135 significantly enriched GO terms were obtained for target genes of DE miRNAs in CY compared with CL, in which vesicle-mediated transport (*p*=6.04E-06), endoribonuclease activity (*p*=1.77E-3) and Golgi apparatus (*p*=6.08E-05) were the top listed GO terms involved in biological process, molecular function and cellular component, respectively (Fig. 4A, Supplementary Table S6). Meanwhile, 88 terms were significantly enriched for target genes of DE miRNAs in CY compared with YK, in which regulation of protein kinase activity (*p*=6.51E-05), nucleoside-triphosphatase activity (*p*=6.58E-05) and postsynapse (*p*=8.40E-05) were the top listed GO terms involved in biological process, molecular function and cellular component, respectively (Fig. 4B, Supplementary Table S7). KEGG enrichment revealed 165 significantly enriched pathways for target genes of DE miRNAs between CY and CL, in which Pathways in cancer (*p*=5.05E-22), MAPK signaling pathway (*p*=8.41E-20) and Axon guidance (*p*=9.99E-16) were the top listed three pathways (Fig. 4C, Supplementary Table S8). Meanwhile, 201 pathways were significantly enriched for target genes of DE miRNAs between CY and YK, in which Pathways in cancer (*p*=6.41E-29), MAPK signaling pathway (*p*=3.44E-28) and Axon guidance (*p*=2.01E-18) were the top listed three pathways (Fig. 4D, Supplementary Table S9). In the cell cycle and P53 signaling pathway presented in Fig. 5, multiple genes (such as *CDK 2/4/6*,* HDAC* and *E2F*) were targeted by bta-miR-34c. CDK 2/4/6 could repress the expression of Rb by phosphorylation, which resulted in the disassociation of Rb with E2F1, E2F2 and E2F3. In return, the DP-1,2 integrated with E2F1, E2F2 and E2F3 could contribute to the synthesis of S-phase protein, thus promoting the transition of G1 to S.

### RT-qPCR validation of DE miRNAs and their target genes

**Figure 6:**
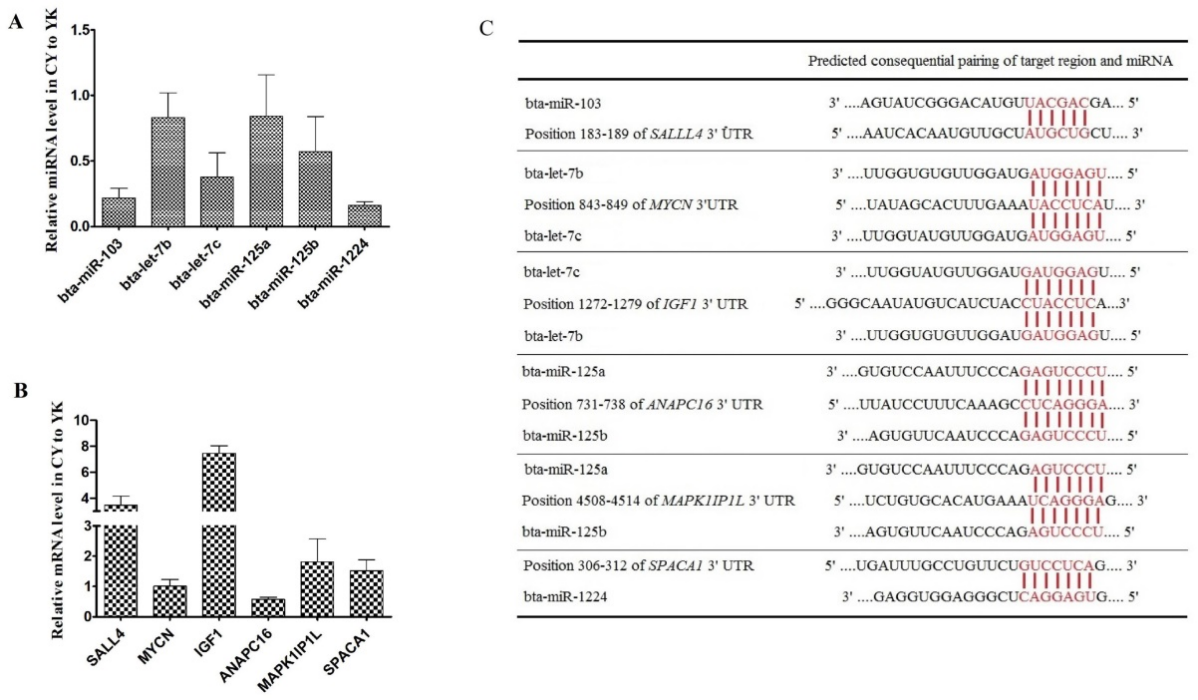
Distinct expression patterns and the binding sites of miRNAs (A, C) and their target genes (B, C).

To validate the expression level of DE miRNAs, stem-loop RT-qPCR was performed on 6 miRNAs (bta-miR-103, bta-let-7b/c, bta-miR-125a/b and bta-miR-1224) and the corresponding target genes. As shown in Fig. 6A, comparison of miRNA expression revealed that the all the miRNAs selected were down-regulated in CY with respect to YK, which was fully consistent with their expression patterns obtained from microarray data. In addition, 6 target genes (*MYCN*, *SAL4*, *IGF1*, *ANAPC16*, *MAPK1IP1L* and* SPACA1*) of these miRNAs were selected to validate their expression levels (Fig. 6B). The expression levels of all the target genes were up-regulated in CY compared with YK except the down-regulation of *MYCN* and* ANAPC16* which might attribute to the fact that a specific gene could been targeted by multiple miRNAs (Fig. 6C).

### Bta-miR-34c over-expression inhibited *CD4*, *CDC25A*, *HDAC1* and *SIRT1*

**Figure 7:**
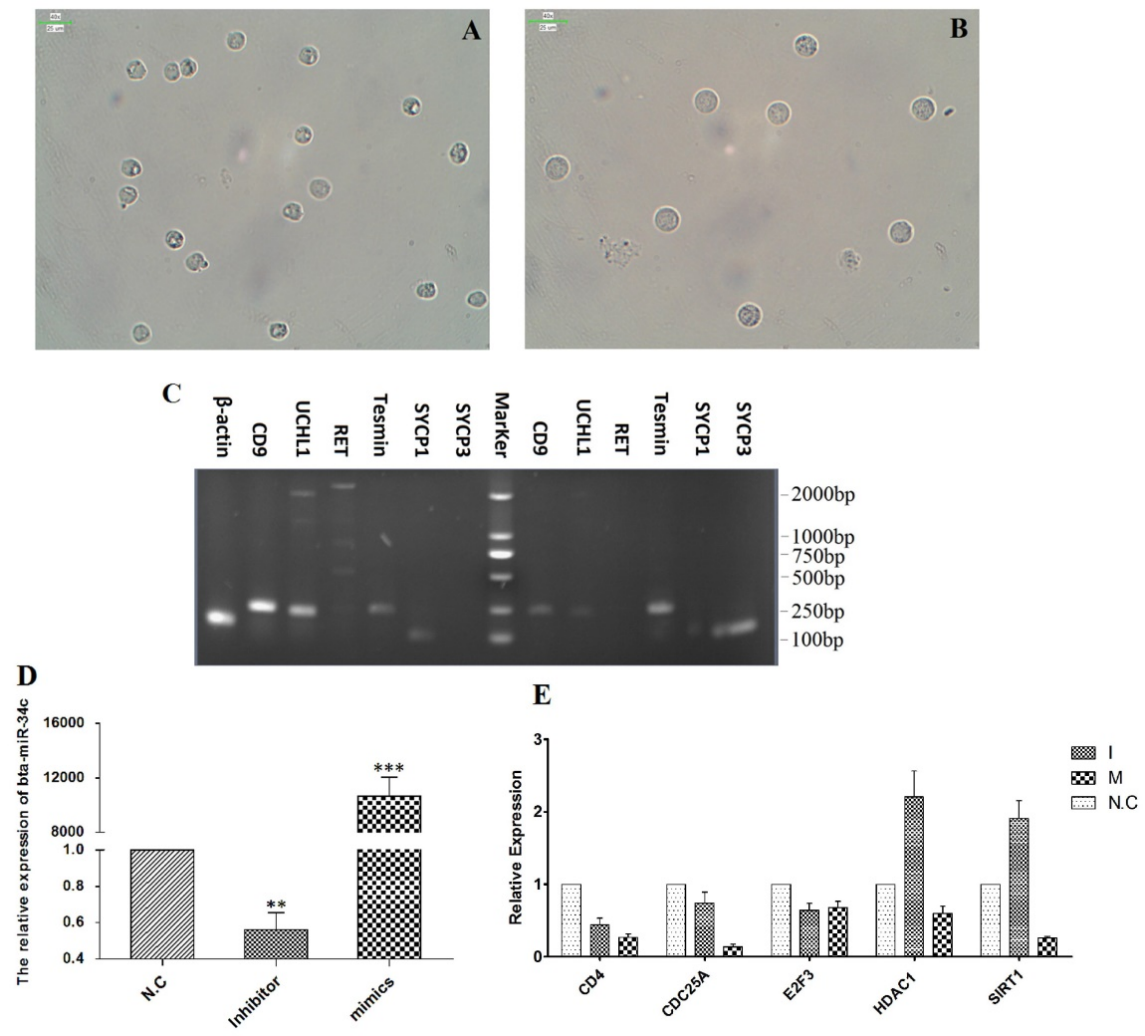
** Effects of bta-miR-34c over-expression on the spermatogonia of YK.** Spermatogonia **(A, C left)** and spermatocytes **(B, C right)** were isolated from YK; the expression of bta-miR-34c **(D)** and their target genes **(E)** after YK spermatogonia transfected with miRNA mimics/inhibitors. I: bta-miR-34c inhibitors; M: bta-miR-34c mimics. Scale bars = 25 µm.

To validate the regulation roles of bta-miR-34c in the initiation of the meiosis, bta-miR-34c mimics and inhibitors were transfected into the spermatogonia of YK. Spermagonia and spermatocytes were obtained from YK by STA-PUT and were identified by the corresponding mark genes (Fig. 7A-C). The qPCR results presented that the bta-miR-34c efficiently transfected into the spermatogonia and over-expression of bta-miR-34c down-regulated the expression of *CD4*, *CDC25A*, *HDAC1* and *SIRT1* (Fig. 7D-E).

## Discussion

Isolation and enrichment of spermatogenic cells at stage-specific development are critical for expanding the knowledge of spermatogensis and for the basic researches on cell biology and application. In previous studies, much progress have been achieved to successfully obtain sub-types of male germ cells with high purity and viability in bovine by employing such methods as differential plating, FACS and MACS 26-28. Comparing the efficiency and practice of different enrichment method for bovine type A spermatogonia revealed that differential plating was a better method of enriching large numbers of type A spermatogonia required for germ cell transplantation, while MACS or FACS could provide highly purified type A spermatogonia suitable for *in vitro* long-term culture 28. However, there is a lack of information for the isolation and enrichment of spermatocytes in bovine due to the various limitations using the methods described above such as lacking the appropriate surface markers for the application of FACS and MACS or failing to efficiently eliminate Sertoli cells and myoid cells by differential plating. Recently, STA-PUT has been successfully applied to the purification of spermatogonia, pachytene spermatocytes and round spermatids in human 8, pig 29 and mice 30. In the present study, we employed the STA-PUT to obtain the mixture of spermatogenic cells containing spermatogonia and primary spermatocytes from CL, YK and CY, which were verified by the morphological and phenotypic identification. *CD9* and* ubiquitin carboxyl-terminal hydrolase 1* (*UCHL1*) have been used for the identification of undifferentiated spermatogonia in bovine 31,32, while the expression of* RET* as SSCs specific marker in bovine has not been fully evaluated. It's not clear why the spermatogenic cells from CL, YK and CY were negative to the marker *RET*, but the reason for this result might be attributed to the differences among species and the effect of age on the population of cells expressing SSCs specific markers in bovine after puberty 33. *Tesmin*, an early marker for male germ cell differentiation, was expressed in a stage-specific manner in all stages of meiotic prophase Ⅰ except preleptonema and leptonema 34, which might account for the relatively lower expression in CL compared with YK. The absent expression of *Tesmin* and *SYCP1* in CY might result from the fact that spermatogenic arrest occurred at an earlier stage of spermatogonia differentiation as observed in Fig. 1. Collectively, these results demonstrated that these cells could be used for the identification of DE miRNAs which might provide valuable information on their regulatory roles involved in the process of spermatogonia differentiation to spermatocytes.

Continuous sperm production in the testis is dependent on the self-renewal of SSCs to maintain their own appropriate population and the differentiation of SSCs to provide adequate numbers of progenitor spermatogonia undergoing the subsequent processes in spermatogenesis. Some miRNAs have been identified to regulate these processes. Highly expressed miR-10b enhanced the proliferation of mouse SSCs by targeting *Klf4,* a pleiotropic zinc finger transcription factor involved in the differentiation and cell-cycle control of several cell types in the mouse 35,36, while the knockdown of miR-10b significantly increased the apoptosis of SSCs 37. MiR-106a and miR-20 were found over-expressed in mouse SSCs to promote the renewal of SSCs at the post-transcriptional level by targeting* STAT3* and* Ccnd1* 38. In the present study, the down-regulation of bta-miR-10b and bta-miR-106a in CY compared with YK could contribute to the impaired proliferation of SSCs and result in the decreased number of spermatogonia observed in the testis of CY (Fig. 1) 14. On the other hand, retinoic acid (RA) is critical in spermatogenesis to modulate the first differentiation of spermatogonia from A_al_ to A_1_ and the entry to meiosis 39. The up-regulation of the X chromosome-clustered miR-221/222 induced by niche growth factor including GDNF, FGF2 and CSF-1 contributed to the maintenance of the mouse undifferentiated THY1^+^ spermatogonia and its proliferation until the exposure to RA signaling, which subsequently promoted conversion to a KIT^+^ state by down-regulating miR-221/222 abundance 40. The induction of miR-let-7 families through the repression of the pluripotency factor LIN28 by RA blocked spermogonial proliferation and promoted the RA-induced spermatogonial differentiation by targeting *Ccnd1, Colla2* and *Mycn* 41. However, the down-regulation of miR-let-7 families in mice functioned as an upstream mechanism for the up-regulation of IGF1/IGE1R which contributed to the activation of ERK1/2 and PI3K as a downstream mechanism of promoting the differentiation of spermotogonia to primary spermatocytes 42. Therefore, the regulatory roles of bta-miR-221/222 and bta-let-7 families in specific development-stage of spermatogenesis remained to be elucidated. In addition, it was worthwhile to note that several miRNAs (bta-let-7a-5p, bta-let-7b/c, bta-miR-125 and bta-miR-23a) were down-regulated in CY simultaneously compared with CL and YK. These miRNAs have been shown significantly up-regulated with testicular RA intervention by administration of CYP26B1 inhibitor and all-trans-RA in dogs 43, suggesting these miRNAs play a vital role in the regulation of RA-induced differentiation of spermatogonia, which was consistent with our finding that the spermatogenic arrest of CY started from the early differentiation of spermogonia 14. However, further studies were needed to elucidate the specific roles of these miRNAs involved in the spermatogenic arrest of CY.

Meiosis, an intricate phase in spermatogenesis, must be carefully regulated and any mistake in this process leads to spermatogenic arrest. Previous studies have revealed that the meiotic stages of lepto/zygotene were mostly transcriptionally inert, which suggested that different post-transcriptional regulations were involved in gene expression 44,45. However, few miRNAs have been identified to be critical in this process. Upon the initiation of meiosis, the miR-449 cluster and miR-34b/c functioned redundantly in down-regulating the activities of the E2F-pRb pathway in murine testes 46. The active E2F-pRb pathway appeared to be related with the spermatogonial proliferation during the mitotic phase in spermatogenesis, while the lower E2F-pRb pathway activity probably contributed to the transition of male germ cells from the mitotic cycle to the meiotic program and prevented meiotic male germ cells from undergoing apoptosis 47-49. In miR-34bc^-/-^ and miR-449^-/-^ mice, a high incidence of apoptosis in pachytene stages of meiosis and a specifical reduction in the number of germ cells after pachytene stages were observed, which led to sterility due to the production of abnormal spermatozoa with reduced motility 50. Spermatocytes were most prone to apoptosis due to any chromosomal mishaps resulted from double strand breaks for undergoing crossover/homologous recombination during the meiotic phase, which could trigger the meiotic checkpoint mechanism and led to spermatocyte apoptosis in p53-dependent and -independent pathways 51-54. In the present study, qPCR results revealed that the over-expression of bta-miR-34c by transfection of miRNA mimic/inhibitor into spermatogonia of YK could block the transition of G1/S by repressing the expression of CDKs and promoting the formation of E2F-Rb (Fig. 5 and Fig. 7). In addition, TUNEL-POD revealed that the most germ cells undergoing normal apoptosis were meiotic cells in YK, which might result from the precise regulation of homeostasis of different cell types to maintain the constant number of meiotic cells in testis 55. However, almost all the apoptotic germ cells were spermatogonia in CY and the apoptosis was probably aggravated in the spermatocytes of CY, which was consistent with our previous finding that spermatogenic arrest of CY may have gotten aggravated during meiosis 14. Therefore, the down-regulation of bta-miR-449a in CY might contribute to the failure of the transition from mitosis to meiosis and the significant reduction in the number of spermatocytes. In addition, it would be important to determine whether there were the redundancy regulation roles between bta-miR-34c and bta-miR-449a in the spermatogenic arrest of CY.

## Supplementary Material

Supplementary figures.

Table S1.

Tables S2-S9.

## Figures and Tables

**Table 1:** Summary of DE miRNAs from the spermatogonia and spermatocytes of CY compared with CL and YK, respectively

MiRNA name	CY VS CL				CY VS YK				Number of targets
	CY_NS	CL_NS	Fold change	Up/Down regulated	CY_NS	YK_NS	Fold change	Up/Down regulated	
bta-let-7a-5p	9.00E-01	6.04E+00	2.84E-02	DOWN	9.00E-01	9.35E+00	2.86E-03	DOWN	229
bta-let-7b	6.97E+00	9.74E+00	1.46E-01	DOWN	6.97E+00	1.27E+01	1.93E-02	DOWN	64
bta-let-7c	5.50E+00	8.31E+00	1.43E-01	DOWN	5.50E+00	1.07E+01	2.75E-02	DOWN	30
bta-let-7e	7.11E-01	2.42E+00	3.05E-01	DOWN	7.11E-01	7.95E+00	6.62E-03	DOWN	9
bta-miR-1224	2.67E+00	3.91E+00	4.23E-01	DOWN	2.67E+00	4.23E+00	3.39E-01	DOWN	554
bta-miR-125a	1.52E+00	6.87E+00	2.46E-02	DOWN	1.52E+00	8.80E+00	6.46E-03	DOWN	597
bta-miR-125b	1.11E+00	6.39E+00	2.58E-02	DOWN	1.11E+00	9.91E+00	2.25E-03	DOWN	470
bta-miR-151-5p	6.50E-01	2.09E+00	3.68E-01	DOWN	6.50E-01	8.44E+00	4.52E-03	DOWN	840
bta-miR-191	1.29E+00	5.01E+00	7.57E-02	DOWN	1.29E+00	8.77E+00	5.59E-03	DOWN	511
bta-miR-193a-5p	1.31E+00	4.54E+00	1.07E-01	DOWN	1.31E+00	6.59E+00	2.59E-02	DOWN	826
bta-miR-20a	9.81E-01	1.99E+00	4.98E-01	DOWN	9.81E-01	5.35E+00	4.85E-02	DOWN	166
bta-miR-214	1.34E+00	5.56E+00	5.35E-02	DOWN	1.34E+00	8.02E+00	9.70E-03	DOWN	1632
bta-miR-23a	7.21E-01	3.57E+00	1.39E-01	DOWN	7.21E-01	8.99E+00	3.24E-03	DOWN	828
bta-miR-23b-3p	1.30E+00	5.52E+00	5.34E-02	DOWN	1.30E+00	1.10E+01	1.22E-03	DOWN	191
bta-miR-2411-3p	5.56E+00	4.49E+00	2.09E+00	UP	5.56E+00	4.53E+00	2.04E+00	UP	17
bta-miR-24-3p	4.25E-01	4.21E+00	7.24E-02	DOWN	4.25E-01	1.04E+01	1.02E-03	DOWN	1359
bta-miR-2440	1.21E+00	2.97E+00	2.95E-01	DOWN	1.21E+00	4.13E+00	1.32E-01	DOWN	822
bta-miR-2454-5p	2.53E+00	4.16E+00	3.22E-01	DOWN	2.53E+00	4.21E+00	3.12E-01	DOWN	2594
bta-miR-26a	3.25E+00	6.30E+00	1.21E-01	DOWN	3.25E+00	1.09E+01	4.80E-03	DOWN	565
bta-miR-296-3p	7.13E+00	5.77E+00	2.58E+00	UP	7.13E+00	5.69E+00	2.71E+00	UP	553
bta-miR-320a	2.11E+00	6.08E+00	6.39E-02	DOWN	2.11E+00	8.83E+00	9.48E-03	DOWN	798
bta-miR-342	1.18E+00	4.10E+00	1.32E-01	DOWN	1.18E+00	9.23E+00	3.76E-03	DOWN	676
bta-miR-423-5p	1.67E+00	4.10E+00	1.86E-01	DOWN	1.67E+00	5.19E+00	8.74E-02	DOWN	2371
bta-miR-424-3p	6.78E-01	2.79E+00	2.31E-01	DOWN	6.78E-01	6.49E+00	1.78E-02	DOWN	321
bta-miR-455-3p	1.26E+00	6.04E+00	3.64E-02	DOWN	1.26E+00	6.99E+00	1.89E-02	DOWN	744
bta-miR-92a	2.68E+00	6.52E+00	6.99E-02	DOWN	2.68E+00	8.70E+00	1.54E-02	DOWN	67
bta-miR-99b	1.81E+00	4.46E+00	1.59E-01	DOWN	1.45E+00	2.84E+00	3.83E-01	DOWN	64

## Abbreviations

CL: cattle
CY: cattleyak
DE: differentially expressed
DPC: Discontinuous Percoll Centrifugation
FACS: Fluorescent-activated cell sorter
GO: Gene Ontology
KEGG: Kyoto Encyclopedia of Genes and Genomes
MACS: Magnetic-activated cell sorting
miRNA: microRNA
NC: oligonucleotides
qPCR: quantitative real-time PCR
RMA+DABG: Robust Multi-array Average plus Detection Above the Background
SSCs: Spermatogonial Stem Cells
*UCHL1*: *ubiquitin carboxyl-terminal hydrolase 1*
VSUG: velocity sedimentation under unit gravity
YK: yak
