# Supplementary figures and images for: Bovid microRNAs involved in the process of spermatogonia differentiation into spermatocytes
Source: Int J Biol Sci. 2020 Jan 1;16(2):239–50. doi: 10.7150/ijbs.38232 (PMC6949159; doi:10.7150/ijbs.38232)

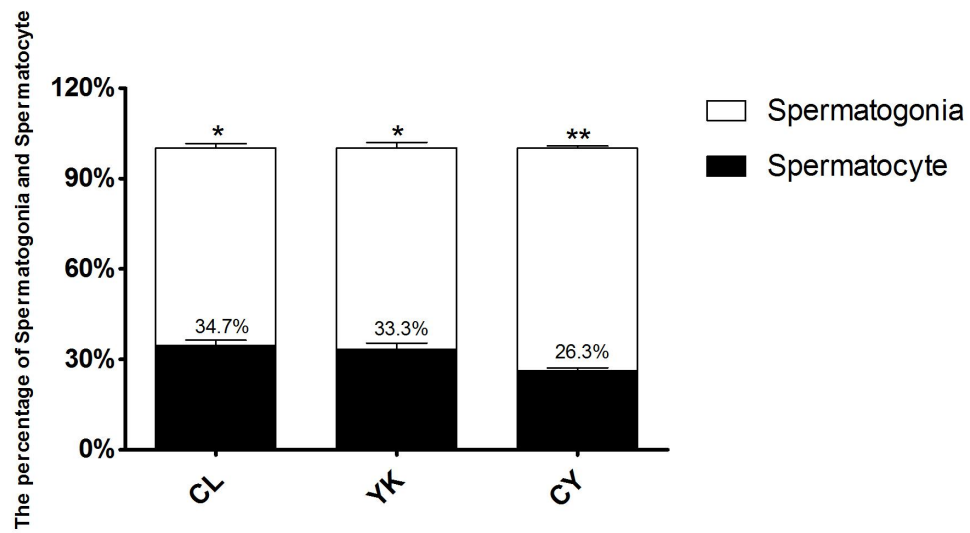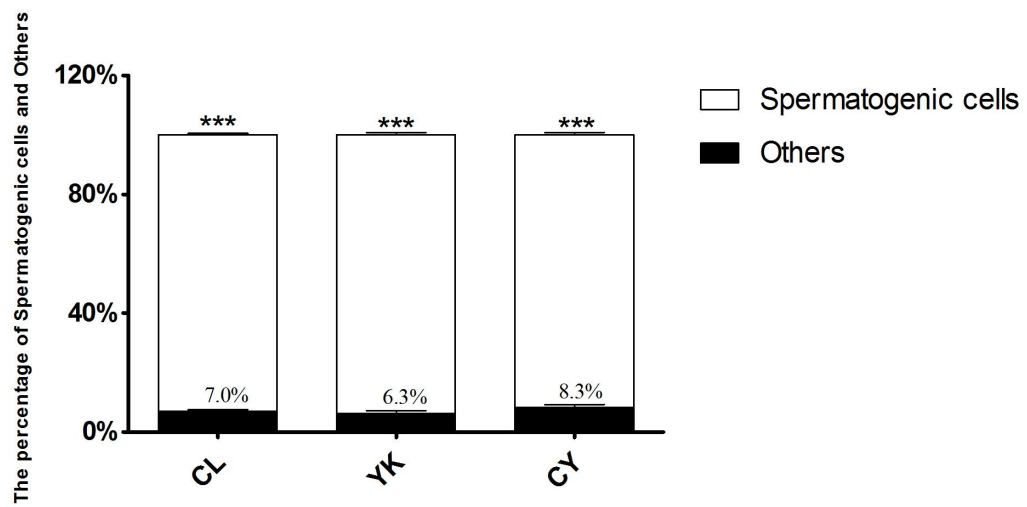

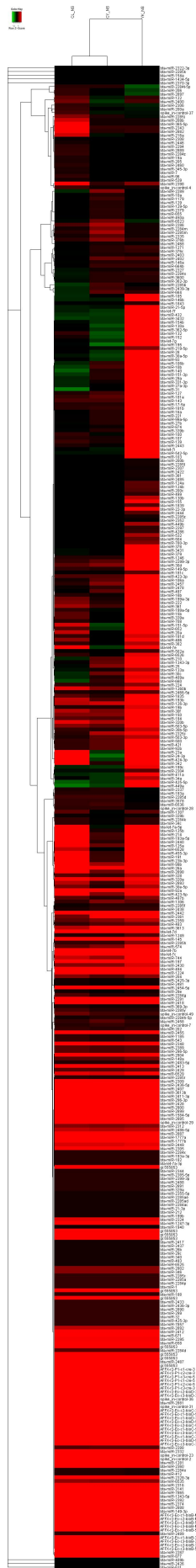

Supplement: Supplementary file 1 — Supplementary figures. [file ijbsv16p0239s1.pdf]
