# Supplementary material for: Bovid microRNAs involved in the process of spermatogonia differentiation into spermatocytes
Source: Int J Biol Sci. 2020 Jan 1;16(2):239–50. doi: 10.7150/ijbs.38232 (PMC6949159; doi:10.7150/ijbs.38232)
Supplement: Supplementary file 2 — Table S1. [file ijbsv16p0239s2.pdf]

| Gene symbol | Stem-loop RT primer (5′-3′) | Forward primer (5′−3′) | Reverse primer (5′−3′) | PCR product (bp) |
|---|---|---|---|---|
| GAPDH | ----- | CATGTTTGTGATGGGCGTGA | GCCAGTAGAAGCAGGGATGA | 250 |
| CD9 | ----- | ATTCGACTCGCAGACCAAGA | TGATCACCTCCTCCTTGTGG | 242 |
| UCLH1 | ----- | GAGTTCGAGGATGGGTCTGT | GGAAAAGGCATCCGTCCATC | 227 |
| RET | ----- | AGATCTCACGAGGGATGCAG | AGGACCACACATCACTCTGG | 240 |
| Tesmin | ----- | TGATTGCTTTGCTAACGGGG | TTGGCCTCATAGCACTCACA | 240 |
| SYCP1 | ----- | ACTTGTGCCAGATCTGCAGA | GCAGTGATACCTGCTTTTCCT | 247 |
| SYCP3 | ----- | CATTCCGGGAAGTTGGCAAA | CAGCTCCAAATCTTTCCAGCA | 214 |
| bta-miR-103 | gtgatccagtcggtccgaggtcagattccgactggatcac TCATAGCCC | gcgAGCAGCATTGTACAG | GTCGGTCCGAGGTCAG | 55 |
| bta-miR-let7b | gtgatccagtcggtccgaggtcagattccgactggatcac AACCACACA | gcgTGAGGTAGTAGGTTG | GTCGGTCCGAGGTCAG | 54 |
| bta-miR-let7c | gtgatccagtcggtccgaggtcagattccgactggatcac AACCATACA | gcgTGAGGTAGTAGGTTG | GTCGGTCCGAGGTCAG | 54 |
| bta-miR-125a | gtgatccagtcggtccgaggtcagattccgactggatcac CACAGGTTA | gcgTCCCTGAGACCCTTT | GTCGGTCCGAGGTCAG | 55 |
| bta-miR-125b | gtgatccagtcggtccgaggtcagattccgactggatcac TCACAAGTT | gcgTCCCTGAGACCCTAA | GTCGGTCCGAGGTCAG | 54 |
| bta-miR-1224 | gtgatccagtcggtccgaggtcagattccgactggatcac CTCCACCTC | gcgGTGAGGACTCGGGAG | GTCGGTCCGAGGTCAG | 53 |
| Bta-miR-34c | gtgatccagtcggtccgaggtcagattccgactggatcac GCAATCAGC | gcgAGGCAGTGTAGTTAG | GTCGGTCCGAGGTCAG | 55 |
| SALL4 | ----- | GGTGAGAAGCCCTTTGTGTG | CTTGGGAAACATCTCGGGGA | 186 |
| IGF1 | ----- | GGAATCGTGGATGAGTGCTG | CCTGCACTCCCTCTACTTGT | 173 |
| MYCN | ----- | CTTCACCATCACCGTTCGTC | GGAGGCTTCGCTCTTGATCT | 178 |
| ANAPC16 | ----- | ATGTTGGAAGACGGCTCTGA | AATCGCCATTCATCTGCCTC | 155 |
| MAPK1IP1L | ----- | TTCCTGAGCTTCCAAGACCA | TGGCCCTGGAGATGGATATG | 152 |
| SPACA1 | ----- | TGGAAGAATGTCGTGGACCA | GGTCTGGTCGTTCGGTAATA | 151 |
| β-actin | ----- | AAGTTCTACAGTGTGGCCGA | GACTGGCCCCTTCTCCTTAG | 150 |
| U6 | ----- | CAGCTGCTTCCTCTAGAGC | CAAATCCCAGAAGTGTGCCA | 100 |
| CDC25A | ----- | TCAAGGGTGCAGTGAACTTG | CTCTCTCACGTATCGGCACA | 155 |
| CDK4 | ----- | GCTTATGGACGTCTGTGCAA | GCAGTTGGCATGAAGGAAAT | 187 |
| E2F3 | ----- | TTTGGCAAGTACCCAAGGAC | GAGGCCAGAGGAGAAAGGTT | 192 |
| HDAC1 | ----- | CTGGGGACCTACGGGATATT | GATCCCCAGACAAGGAGTCA | 186 |
| SIRT1 | ----- | CCAGACCTTCCAGATCCTCA | CTGCAACCTGCTCCAGTGTA | 202 |

**Supplementary Table S1. Primer sequences for PCR of the selected genes and miRNA.**
